# Tung oil as an effective modifier for sulfur polymer cement and its performance in galvanic waste encapsulation

**DOI:** 10.1016/j.heliyon.2020.e03908

**Published:** 2020-05-05

**Authors:** Kamil Banaszkiewicz, Franciszek Czechowski

**Affiliations:** a Faculty of Environmental Engineering, Unit of Technologies of Waste Materials and Soil Remediation, Wroclaw University of Science and Technology, Pl. Grunwaldzki 13, 50-377 Wrocław, Poland; b Faculty of Earth Sciences and Environmental Management, University of Wrocław, Pl. Maksa Borna 9, 50-204 Wroclaw, Poland

**Keywords:** Civil engineering, Chemical engineering, Environmental engineering, Waste treatment, Environmental management, Waste, Tung oil, Sulfur polymer cement, Galvanic waste, Stabilization/solidification, Leaching test, Compressive strength

## Abstract

The data on the performance of sulfur polymer cement crosslinked with tung oil polymerization modifier are presented. Specimens of sulfur polymer cement (SPC) were prepared with different doses of tung oil in amounts of up to 8.85% of the sulfur mass. The obtained SPCs were used as binders to encapsulate two galvanic wastes differing in their toxic metal composition: waste **I** and waste **II** with loadings of approximately 25 and 50% of the composites' mass, respectively. For comparative purposes, appropriate samples of the SPCs and their composites with galvanic wastes were obtained using very similar doses of dicyclopentadiene sulfur modifier. Waste II was also encapsulated using SPC, in which a mixture of tung oil and dicyclopentadiene in a 1:1 weight ratio was used as the modifier. Crosslinking of the tung oil to the SPC matrix was assessed by FT-IR. The obtained SPCs and their composites with galvanic wastes were characterized by SEM and tested for water sorption capacity, compressive strength and metal leaching toxicity using TCLP and EN standards. The effectiveness of the tung oil binding to the SPC network was evidenced by the complete disappearance of methine C–H stretching vibrations at 3010 cm^−1^ and the double bond –C=C– wagging vibrations at 990 cm^−1^ in the FT-IR spectrum after processing with sulfur. SEM observations revealed that all the specimens prepared with dicyclopentadiene had a glassy-like fracture surface and also showed fewer cavities and defects in cements and composites when compared to their counterparts prepared with tung oil. The water sorption capacities of all the specimens were below 1%, where the values of those prepared with the tung oil were two to three fold higher than the values of their counterparts prepared with dicyclopentadiene. The pH of the TCLP leachates was in the range of 2.75–2.98, and a decreasing trend in the pH value was found with an increasing modifier dose. The TCLP leachate pH from the waste **I** monoliths with dicyclopentadiene were generally lower by 0.1–0.35 when compared to the corresponding monoliths with tung oil. The toxic metals immobilization order revealed from the TCLP test (leachate pH around 2.85) is Cd > Sr ≥ Zn > Cu > Ni > Cr > Pb, while the resulting order from the EN test, due to a higher leachate pH of about 5.9, follows Cd > Pb > Zn > Cu ≥ Ni > Sr > Cr. An increased tung oil dose from 2 to 8.85% enhanced the SPC compressive strength by three to four fold, while the same increase of the dicyclopentadiene dose led to an increase of this parameter for less than two fold. The addition of galvanic wastes to the SPCs resulted in a further increase in compressive strength for the corresponding SPC samples.

## 1 Introduction

Various methods are used for preventing heavy metal leaching from industrial wastes into the natural environment. In this respect, the immobilization by chemical stabilization of toxic metals contained in wastes to less soluble forms does not protect their deterioration in weathering processes. Numerous earlier studies have shown that physical entrapment of such wastes through encapsulation or solidification diminishes their exposure to weathering and substantially reduces leaching. This was demonstrated for wastes containing, among others toxic metals, Zn, Ni, Cu, Cr and Pb, where conventional Portland cement was shown to be effective as a binding material [1, 2, 3]. However, the production of Portland cement is an energy consuming process that is associated with significant greenhouse gas emissions in an amount of about one tonne of carbon dioxide per tonne of obtained cement [4]. Therefore, it has become necessary to search for other materials that could be used as an effective binder and that could replace hydraulic Portland cement. Over the last few decades, a lot of interest was focused on sulfur based cements and their use for the above purposes [5, 6, 7]. However, the presence of sulfur alone in such a composition caused limitations in strength and durability under repetitive freeze-thaw cycling [8]. This is due to the fact that during cooling of the prepared liquid cement, sulfur first crystallizes to monoclinic sulfur (β-sulfur). Then, at a temperature below 95.3 °C, it undergoes allotropic phase transformation to orthorhombic sulfur (α-sulfur), which is associated with a shrinkage in volume that causes internal stresses in the formed cement structural network [9]*.* These weak characteristics were reduced by the addition of dicyclopentadiene (DCPD) or other synthetic unsaturated organic modifiers like cyclopentadiene and dipentene. These chemicals strengthen the crosslinking of the sulfur cement matrix during the hot-mix step of cement preparation [10, 11, 12, 13, 14, 15]. Modified sulfur polymer cements (SPCs) are cost-effective materials when compared to Portland cement, and their production is therefore less harmful to the environment. It is also a beneficial process from an energy and environmental point of view, involving the use of sulfur being a by-product produced in large quantities in the petrochemical industry. Such SPCs with organic polymerization modifiers show increased durability and have superior resistance to acidic and salty environments [16]. The documented performance of such binders met the requirements for binders used for hazardous waste solidification and for storage in extreme environmental conditions [17, 18, 19]. Thus, the replacement of Portland cement with SPCs is significantly beneficial to the environment regarding the simultaneous management of waste sulfur and hazardous wastes. Moreover, this also contributes to carbon dioxide sequestration. The further advantageous properties of SPCs based on TO used as binding materials are the ease of obtaining a homogenous mixture with waste components, and the formation of solid monoliths of a desired shape. Furthermore, the production of SPCs can last throughout the year because it is not dependent on negative climate temperatures. The obtained products also attain full strength within a few hours.

However, conventional SPC modifiers used until now are harmful organic solvents. Therefore, developing SPC binders using modifiers from natural bio-resources may attract the attention of industry and academia. Such materials modified with renewable bio-origin crosslinkers have already been developed [20, 21].

The paper presents data on the performance of SPC modified with various doses of natural non-edible tung oil (TO) as an environmentally friendly crosslinking agent, and also its usefulness for the stabilization/solidification of hazardous galvanic waste. Such binder has an added value for the environment because the sulfur polymerization modifier is not a synthetic compound, but instead an easily available green natural compound. The usefulness of SPC based on TO as a binder for hazardous wastes was assessed on the basis of physicochemical testing data for the obtained monoliths containing galvanic waste. Its performance was compared with data on the counterpart composites in which SPC was modified with DCPD and a mixture of TO + DCPD in a 1:1 weight ratio.

## 2 Materials and methods

### 2.1 Reagents for the preparation of sulfur polymer cement

Chemical compounds used for the preparation of sulfur polymer cement were purchased from chemical reagent stores. Sulfur powder (CAS 7704-34-9) with 99.85% of elemental sulfur was purchased from Chempur, Poland. Both modification reagents used to prepare the SPC were supplied from Merck: dicyclopentadiene (CAS 77-73-6) with a purity ≥90%, and tung oil (CAS 8001-20-5) composed of triglyceride esters with *α-*eleostearic acid (cis-9,trans-11,trans-13-octadecatrienoic acid), linoleic acid, oleic acid and palmitic acid, where the content of α-eleostearic acid ester was approximated at 84% w/w.

### 2.2 Galvanic waste

Two batches of galvanic sludge collected at different times from a Lower Silesian galvanizing plant (SW Poland) specializing in galvanic bethanization, nickel plating and passivation in trivalent chromium, were used for the experiments. This was due to limited material being available at the time. Both the sludges were watered above 60%. In the further text their dry materials are assigned as waste **I** and waste **II**. These wastes differed in the composition of the main metals associated with electroplating (Cr, Cu, Zn and Ni, see Table 1). For waste **I** analysis of all the contained metals was performed, while for waste **II** the content of only the main metals was determined. The SPC composites contained around 25% w/w of waste **I**, or around 50% of waste **II** in the monoliths. According to the United States Code of Federal Regulations (CAR), the sludge from electroplating with wastewater treatment operations is classified as hazardous waste - F006 [22]. Similarly, such waste is classified as hazardous according to Polish regulations, where it is included in the waste catalogue - 19 08 13 [23].

**Table 1:** Concentration of heavy metals in galvanic wastes and their leachates from leaching tests.

Metal Leachate pH	Metal concentration in dry waste	Metal concentration in leachate		
		TCLP test	EN test	
	(mg/kg dry wt.)	(mg/L)	(mg/kg dry wt.)	(mg/L)
**Galvanic waste I**				
Cr	166870	75.4	0.5	0.05
Cu	104484	536	137	13.7
Zn	82674	825	3060	306
Ni	5376	13.0	33.7	3.37
Pb	2901	0.17	0.2	0.02
Cd	55	0.54	1.2	0.12
Sr	113	1.46	18.5	1.85
Leachate pH	-	4.53	5.81	

**Galvanic waste II**				
Cr	78075	209	21	2.1
Cu	54712	460	318	31.8
Zn	37939	528	6550	655
Ni	2196	17.3	216	21.6
Leachate pH	-	4.38	5.46	

### 2.3 Preparation of sulfur polymer cement and its composites with galvanic waste

The sulfur polymer cement (SPC, sulfur binder) was prepared from elemental sulfur and the polymerization modifiers: (i) tung oil (TO), (ii) dicyclopentadiene (DCPD) and (iii) a blended mixture of () in the weighted proportion of 1:1. A specified amount of sulfur placed in a cylindrical tube with an internal diameter of 2 cm was melted in a glycerin bath, heated up to 140 °C and intensively stirred using a mechanical stirrer. At this temperature the modifying reagent (i), (ii) or (iii) was added to the molten sulfur at a rate of 5 mL⋅min^−1^⋅kg^−1^ up to the final doses of around 2, 4, 6 or 8% of sulfur mass (% of sulfur mass = %sm). After completion of adding the modifiers, the resulting molten SPC was stirred at a temperature of 135–140 °C for a further 20–30 min. Composite with waste was obtained by further addition of the dry galvanic waste **I** or **II** to the molten SPC in the form of powder with a granulation of less than 0.5 mm. The final content of wastes **I** or **II** was 25 or 50% of the composite weight, respectively. Continuous homogenization by mixing was performed during the addition of waste and lasted for approximately 10–20 min. It should be noted, that in the case of SPC based on tung oil, during the addition of waste (at 135–140 °C), the formation of a gel-like (slightly rubbery) consistency was observed. This phenomenon hindered an even distribution of waste in the whole volume of the prepared samples. They were then degassed by vibration on a vibrating table for about 5 min after which the tubes were cooled at room temperature. The thus obtained monoliths of solidified SPC, and its composites in the form of cylindrical disks with a diameter of 2 cm and a height of 4 cm, were used to assess their properties and effectiveness in immobilizing toxic metals from galvanic waste.

### 2.4 Infrared spectroscopy

Fourier transformed infrared spectra (FT-IR) were obtained with the use of a Nicolet Impact 400 spectrophotometer linked with a computer data processing system. Transmittances of TO and SPC based on TO modifier were taken within the wavelength ranging from 4000 to 400 cm^−1^ with 64 scans and 4 cm^−1^ resolution in KBr pellets prepared with 1 mg of the sample and 40 mg of KBr.

### 2.5 Scanning electron microscopy

The surface image of the solidified specimens was obtained from a VEGA TESCAN scanning electron microscope. To improve the SEM imaging of the specimens, they were sputtered with gold prior to observation.

### 2.6 Water absorption by immersion

Determination of the water absorption capacity involved a stepwise submersion of the solidified monoliths in water at room temperature, and the monitoring of the water uptake (wet mass) and weight loss (dry mass) at defined time intervals. In this method, the monoliths were placed in a container and flooded with water to a quarter of their heights. The samples were kept in this state for 2 h. Then, the level of water was raised to a half of the sample height. After another three hours the water level was further raised to three-quarters of the solidified specimens' height. The submerged samples were left for a further 19 h. Finally, the samples were completely immersed in water. The upper surface of the samples was situated about 2 cm below the water level. With the time, after every 24 h of the samples immersion in water, they were taken from a container and weighed on an electronic balance with an accuracy of ±0.01 g. The weighing was repeated until a constant wet weight of the sample was achieved. Water absorption (W_A_) was calculated as a percentage of the increase of the monolith's mass according to Eq. (1):

(1) $$W_{A}=\frac{M_{b}-M_{a}}{M_{a}}\times 100\text{\%}$$

where: M_a_ – is the mass of the dry monolith, and M_b_ – is the mass of the monolith with absorbed water.

### 2.7 Unconfined compressive strength

The compressive strength of the SPC and its monoliths with galvanic wastes **I** and **II** was determined after 24 h of their preparation. The cylindrical specimens used for this purpose (20 mm in diameter and 40 mm in height) were much smaller than those required in the standard for such materials [24]. This was because of the limited mass of the galvanic wastes available for testing. The compressive strength results for the non-normative monoliths of SPC based on TO modifier and its composite with waste show a comparative value when confronted to the results on similarly shaped monoliths based on conventional DCPD sulfur polymerization modifier. The results obtained are the average values of two measurements for the corresponding monolith.

### 2.8 Leaching test

Leaching tests were carried out in accordance with the TCLP [25], as well as with the procedure described in EN 12457-4:2002 [26] – the obligatory standard in EU countries. In the TCLP test, a sample of granulation <10 mm was extracted with a glacial acetic acid buffer in distilled water (pH = 2.88 ± 0.05, ratio of liquid/solid phase = 20/1 L/kg). After 18 h of shaking on a rotary shaker, the extraction liquid was filtered off through a 0.45 μm pores grade filter and the pH of the resulting leachate was measured. The leachate was then analyzed to determine the content and concentration of heavy metals. In the EN test, the sample with granulation <10 mm was extracted for 24 h with distilled water (ratio of liquid/dry solid phase = 10/1 L/kg), and then the leachate was filtered to remove all suspension and solid particles. It was also analyzed for the content of heavy metals and its pH value was measured.

## 3 Results and discussion

### 3.1 Heavy metals in galvanic wastes and their leachates from leaching tests

The galvanic wastes used for encapsulation in the prepared SPCs contained heavy metals: Cr, Cu, Zn, Ni, Pb, and others (Table 1). Their assignment to the type of waste is classified by the susceptibility of the metals' mobilization, which reflects the toxicity of the waste under disposal conditions. For this purpose, assessment of the metals' mobilization from the galvanic wastes **I** and **II** was achieved with the use of leaching tests. The concentrations of heavy metals in wastes **I** and **II,** as well as in their leachates, are presented in Table 1. The concentrations were obtained from tests carried out in accordance with the toxicity characteristic leaching procedure – TCLP [25], as well as EN 12457-4:2002 (EN test) [26].

The TCLP test showed a high concentration of Cr in the leachates from raw waste **I** and **II** – 75 mg/L and 209 mg/L, respectively. Both values exceeded the 5 mg/L limit defined in 40 CFR 261.24, which classifies such wastes as hazardous [27]. The concentrations of Cd (0.54 mg/L) and Pb (0.17 mg/L) in the waste **I** leachate were below regulatory limits (Cd = 1 mg/L and Pb = 5 mg/L). Despite the high concentration of Cu (536 and 460 mg/L, respectively) and Zn (accordingly 825 and 528 mg/L) in the waste **I** and **II** leachates, they do not indicate hazardous wastes because the US EPA regulatory does not regulate their concentration limits for the classification of solid hazardous waste.

The EN test was carried out to assess the possibility of raw waste disposal at hazardous waste landfills. The criteria established for acceptable hazardous waste at landfills include permissible leaching values for harmful heavy metals, which are for: Cr – 70 mg/kg dry wt., Cd – 5 mg/kg dry wt., Ni – 40 mg/kg dry wt., Cu – 100 mg/kg dry wt., Pb – 50 mg/kg dry wt. and Zn – 200 mg/kg dry wt [28]. In the leachate from waste **I**, the concentration of Cu (1.3 times), and especially Zn (15 times), exceeded the limit values, while for waste **II** the concentrations of Cu, Zn and Ni in the leachate were over 3, 32 and 5 times higher than the permitted levels, respectively. Much higher concentrations of respective metals in the leachates from waste **II**, when compared to waste **I**, result from the lower pH of the leachate from waste **II**. Thus, the results of the leaching tests showed that the above wastes cannot be disposed of in hazardous waste landfills. Therefore, a rational alternative to reducing their negative impact on the environment at the place of storage is their physical immobilization by encapsulation.

### 3.2 Composition of sulfur polymer cements and composites with galvanic wastes

The detailed composition of the obtained SPCs and galvanic waste composites is given in Table 2. The crosslinking modifiers used to prepare the SPCs were bio-origin TO and synthetic DCPD.

**Table 2:** Composition of sulfur polymer cements and monoliths with galvanic wastes.

Assigned sample symbol∗	Sulfur	Modifier		Galvanic waste		Modifier content of sulfur mass
		Tung oil	Dicyclopentadiene	Waste I	Waste II	
	(content in solidified monolith, % w/w)					(%sm)
S	100	-	-	-	-	-
S-**I**25	75.36	-	-	24.64	-	-
ST2	97.93	2.07	-	-	-	2.12
ST4	95.98	4.02	-	-	-	4.19
ST6	94.02	5.98	-	-	-	6.37
ST8	92.03	7.97	-	-	-	8.67
SD2	97.90	-	2.10	-	-	2.14
SD4	95.94	-	4.06	-	-	4.23
SD6	93.97	-	6.03	-	-	6.42
SD8	92.02	-	7.98	-	-	8.67
ST2-**I**25	73.44	1.98	-	24.57	-	2.70
ST4-**I**25	72.32	3.02	-	24.66	-	4.18
ST6-**I**25	70.78	4.57	-	24.65	-	6.46
ST8-**I**25	69.28	6.07	-	24.64	-	8.77
SD2-**I**25	73.50	-	1.97	24.52	-	2.68
SD4-**I**25	72.22	-	3.14	24.64	-	4.34
SD6-**I**25	70.77	-	4.59	24.63	-	6.49
SD8-**I**25	69.24	-	6.13	24.63	-	8.85
ST2-**II**50	49.50	1.00	-	-	49.50	2.02
ST4-**II**50	49.02	1.96	-	-	49.02	3.99
ST6-**II**50	48.54	2.92	-	-	48.54	6.01
ST8-**II**50	48.08	3.84	-	-	48.08	7.98
SD2-**II**50	49.50	-	1.00	-	49.50	2.00
SD4-**II**50	49.02	-	1.96	-	49.02	3.99
SD6-**II**50	48.54	-	2.92	-	48.54	6.01
SD8-**II**50	48.08	-	3.84	-	48.08	7.98
S(T + D)2-**II**50	49.50	0.50	0.50	-	49.50	2.02
S(T + D)4-**II**50	49.02	0.98	0.98	-	49.02	3.99
S(T + D)6-**II**50	48.54	1.46	1.46	-	48.54	6.01
S(T + D)8-**II**50	48.08	1.92	1.92	-	48.08	7.98

∗ code of the sample includes the approximate weighted percentage of the tung oil (T) and/or dicyclopentadiene (D) in the sulfur (S), while the number after the hyphen indicates the approximate amount of galvanic waste **I** or **II** in the composite.

Loadings of the galvanic waste in the prepared composites were approximately 25% w/w of waste **I** and 50 % w/w of waste **II**.

#### 3.2.1 Tung oil as the polymerization modifier for SPC

**Figure 1:**
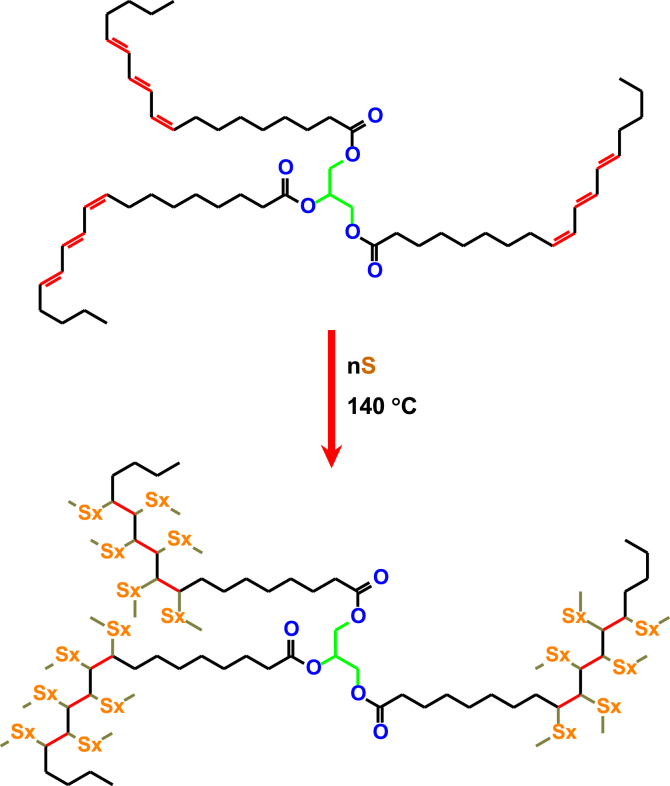
Chemical structure of 1,2,3-α-eleostearoyl glycerol (upper structure) and the product of its reaction with sulfur.

The growing interest in the use of sulfur polymer cements for the purpose of disposing hazardous waste has caused an increase in the interest concerning natural modifiers that enable DCPD or other commonly used synthetic additives to be replaced. Bio-origin TO composed with up to 86% of α-eleostearic acid (cis-9,trans-11,trans-13-octadecatrienoic acid) containing three conjugated unsaturated double bonds per fatty acid chain is a very attractive alternative for this purpose. It is a natural product obtained from tung tree (*Vernicia fordii*) nuts and is widely available at a relatively low cost [29]. The most abundant triglyceride in TO is a triester of α-eleostearic acid with 1,2,3-propanetriol. Its chemical structure is presented in Figure 1 – upper structure. It is well known that such triglycerides with conjugated unsaturated bonds in fatty acid chains readily undergo auto oxidation by air oxygen, where oxidative crosslinking of these double bonds proceeds *via* free radical polymerization forming a multidirectional polymeric network [30, 31, 32].

**Figure 2:**
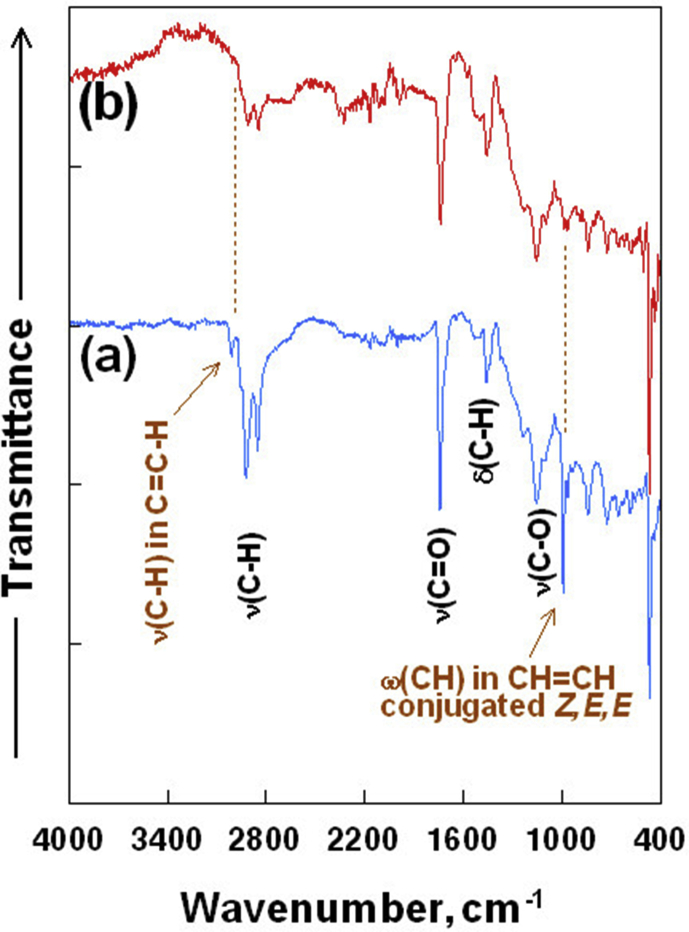
FT-IR spectra of (a) not processed analogue to the ST6 sample, and (b) the ST6 sample.

Similar oxidative polymerization of TO proceeds in the presence of sulfur during the heating stage of SPC preparation through thermally induced free radical polymerization. The fulfillment of the reaction, involving double bonds of the TO components, is illustrated by the FT-IR spectra on the example of the ST6 sample, in which the TO phase constituted 6.42%sm (Figure 2). Spectrum **(a)** in Figure 2 represents a thermally untreated mixture of powdered sulfur with the same TO dose as is in sample ST6, while the spectrum **(b)** refers to the obtained sample ST6. The presence of C=C double bonds is evidenced in the spectrum **(a)** of the sample with unreacted TO by the weak absorbance at 3010 cm^−1^. This is due to the stretching vibrations of the methine moieties υ(C–H) located at the carbon–carbon double bonds C=C-H. It is complemented by the intense absorbance at 990 cm^−1^ and weak absorbance at 964 cm^−1^ that is assigned to the wagging vibrations in the methine groups ω(CH) of cis,trans, trans conjugated double bonds CH = CH [33]. In the spectrum **(b)** of the ST6 sample, virtually complete disappearance of the absorbancies associated with double bonds (υC-H vibrations in C=C-H and ωCH vibrations in CH = CH–CH = CH cis,trans,trans-conjugated) is observed (description of these vibrations on the spectrum are highlighted in brown – Figure 2). This indicates that tung oil has been effectively incorporated into the SPC matrix. The formed three-dimensional polymeric system around the triester units of TO glycerides, and the multiple crosslinking to the sulfur polymeric network in the SPC, promotes the formation of polymeric continuity in the final material (lower structure in Figure 1).

### 3.3 SEM images of the fracture surface

**Figure 3:**
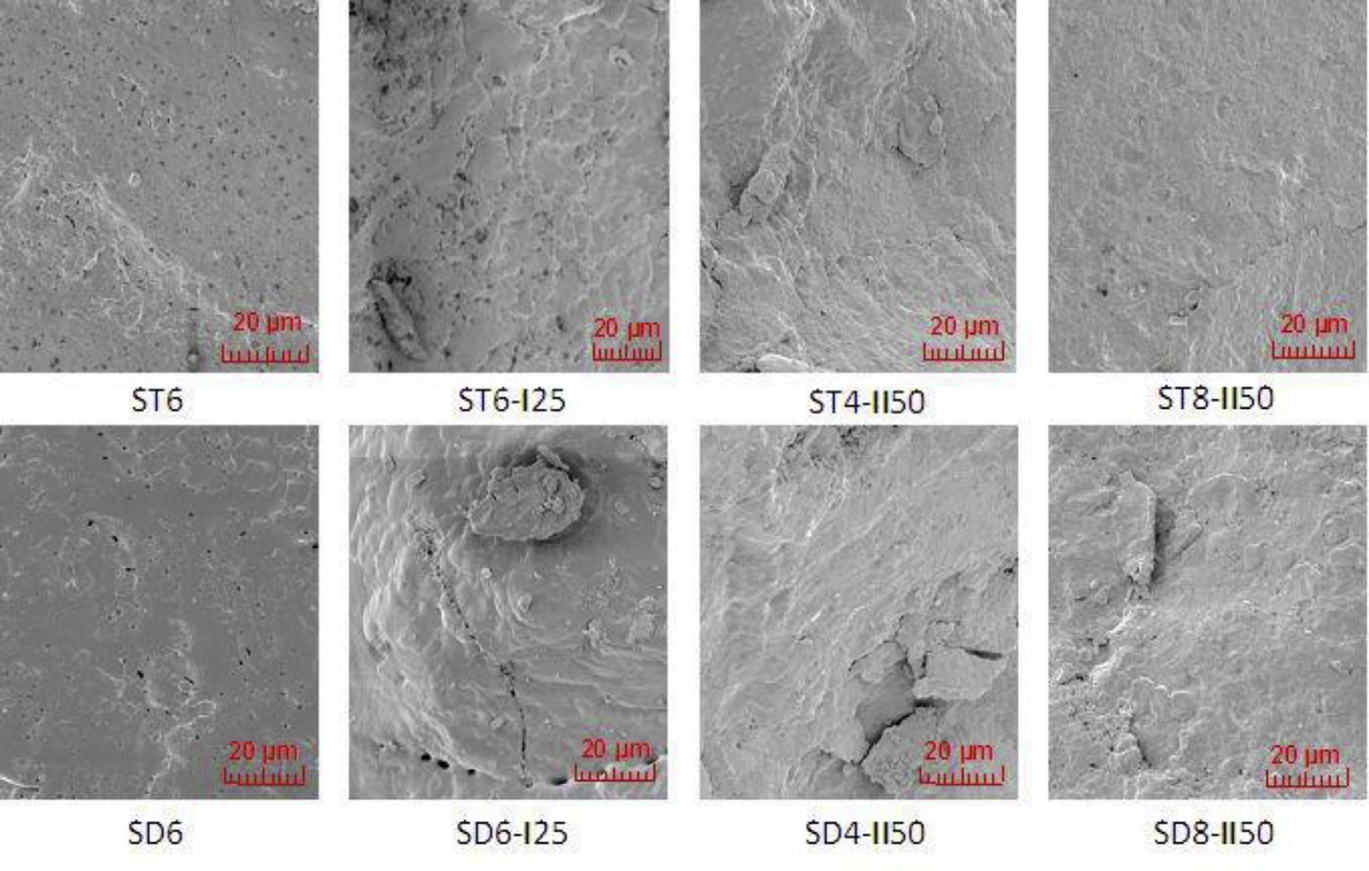
SEM fracture surface micrographs of the selected SPCs and composites with galvanic wastes **I** and **II**.

The fracture surface morphology of the monoliths was characterized by SEM observations. In Figure 3 a typical SEM images of the selected monoliths fracture surface, prepared with TO and DCPD modifiers, are illustrated. These were SPCs, as well as composites with encapsulated galvanic wastes. The illustrations show a more uniform fracture surface of the SPC that is prepared with DCPD (SD6) than the one prepared with the TO modifier (ST6). This is also the case for the composites with the encapsulated galvanic wastes, where a more uniform glassy-like fracture surface, with some fissures, was observed for the respective specimens containing DCPD. In the composites containing TO, the presence of fissures was rarer. The visible surface fissures were presumably formed during crushing of the specimen and do not occur in the interior of the monoliths' matrices. Moreover, dispersion and occlusion of waste particles in the composites containing DCPD is more homogenous and without visible grain clusters. The fracture surfaces of the samples containing TO appear less compact and are with the presence of more vein/bubble openings of around 1–2 μm diameter. They are most likely the result of the incomplete degassing of the specimens prepared with TO, which had a gel-like consistency during preparation. Furthermore, in the composites with TO (for instance, the shown sample ST6-**I**25) the presence of waste grain clusters is observed, which is a result of the more difficult workability of the composites containing TO during the preparation process. Basically, the morphology of the fracture surface is not only dependent on the kind of sulfur modifier, but also on its dose. Along with an increasing modifier dose (TO or DCPD) into the SPCs from 2 to 8%sm, the fracture surface of the solidified monoliths gradually becomes more uniform.

### 3.4 Water absorption capacity by immersion

**Figure 4:**
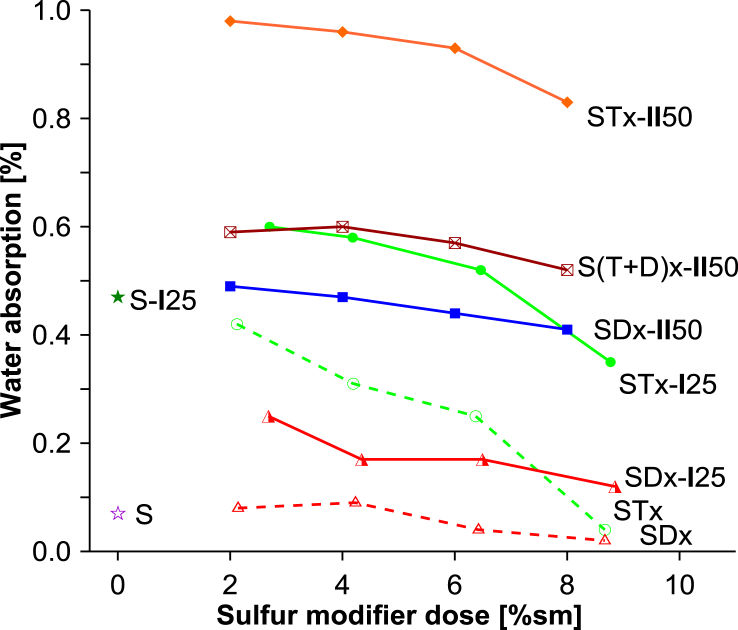
Water absorption capacity of SPC binders and their monoliths with galvanic wastes **I** and **II**. Index ‘x’ in the sample symbol refers to modifier dose percent of sulfur mass (Table 2).

Absorption of water by solidified specimens is a measure of their resistance to water penetration in humid conditions that are close to saturation. Data on the water absorption capacity of monoliths for SPC binders and solidified galvanic waste composites are presented graphically in Figure 4. The general trend in reducing water absorption capacity with increasing the dose of the sulfur modifier was clearly observed for monoliths containing the studied sulfur modifiers – TO or DCPD or blend. All the tested specimens based on the DCPD sulfur modifier (SPCs or composites with wastes) exhibited lower water absorption capacities by 0.3–0.5% than the corresponding specimens prepared with the TO modifier. The lowest water absorption was shown by the solidified SPCs with the DCPD modifier, which was below 0.1% (series SDx in Figure 4). The corresponding values for the SPCs prepared with TO were from one and a half to four times higher (series STx in Figure 4). Regardless of the sulfur modifier, the addition of galvanic waste to the SPCs was accompanied by an increase in water absorption. The addition of waste **I** to the SPCs in an amount of around 25% was manifested by an increase in water absorption to the levels of around 0.2% for the SDx-**I**25 samples and 0.5% for the STx-**I**25 samples. Much higher increases were found for the samples containing 50% w/w of galvanic waste **II**. The highest values were found for the series of samples containing TO and 50% w/w of waste **II** (STx-**II**50; at level close to 1%). Values for the corresponding monoliths containing DCPD (series SDx-**II**50) were twice as low i.e. below 0.5%. Water absorption for the composite series containing the blended modifier (series S(T+D)x-**II**50) was at an intermediate level of about 0.6%. The higher respective values for the SPCs and composites with the TO modifier are probably partly related to their incomplete degassing, and partly due to the lower homogeneity and dispersion of the added galvanic waste particles in the matrices of the SPCs, which allows more extended hydration of mineral waste particles. The monolith specimens were stable upon water immersion, where no changes in their dimensions were recorded.

### 3.5 Compressive strength

**Figure 5:**
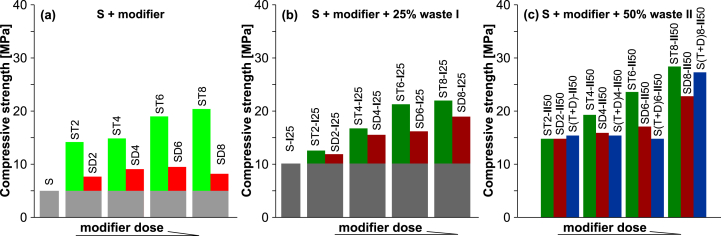
Dependence of the kind of sulfur modifier to the SPC and its dose on the compressive strength of the (a) SPC binders; (b) composites with waste **I**; (c) composites with waste **II**.

Compressive strength is one of the most essential parameter in determining the waste solidification effectiveness in SPC. It is relevant when assessing the structural integrity of monoliths, and should have a sufficient value in order to maintain the stability and shape of the monoliths and to withstand the loading pressures in the disposal place. Moreover, knowledge of the stress that the solidified monoliths can withstand allows the maximum thickness of stored solidified waste to be determined. The compressive strength of the studied SPCs and solidified composite monoliths was determined after 24 h of their preparation. The average value was taken from two measurements for the corresponding monolith. The impact of the studied modifiers and their doses on the compressive strength of the prepared SPCs and composites with galvanic wastes is illustrated on the histogram in Figure 5. The compressive strength value of the control sample (S), i.e. SPC prepared without a modifier, was 4.96 MPa, which was highlighted by the gray bars on histogram **(a)** in Figure 5. Its additionally increased values for the SPCs, due to the added modifier (TO or DCPD) with doses up to 8%sm, are highlighted by light green bars for the TO based SPCs, and red bars for the respective SPCs with DCPD. The gradual increase in compressive strength with a modifier dose from 2 to 8%sm was far greater for the binders with TO (14.2–20.4 MPa) than for the ones with DCPD (7.7–9.5 MPa).

The compressive strength of the control composite with 25% w/w galvanic waste **I** (S-**I**25) was twice as high as for the sample (S) without the addition of waste – compare histograms **(a)** and **(b)** in Figure 5. This indicates the enhancing role of galvanic waste as a filler that increases the compressive strength of the resulting composite. It should be mentioned here, as revealed by the XRD analysis (data are not shown), that the introduced amorphous galvanic waste did not form crystalline minerals with the SPC matrix, but instead remained unchanged as an inert filler. The use of SPCs modified with the studied agents as binders for the preparation of composites with waste **I** resulted in a further increase of compressive strength when compared to the reference sample S-**I**25. Moreover, the strengthening effect is also related to the dose of the modifier. For the composites with TO or DCPD in an amount from 2 to 8%sm, the composites' compressive strength ranges are from 12.6 to 22.0 MPa, and from 11.9 to 19.0 MPa, respectively. However, the additionally increased values attributed to the role of the modifier in the case of the composites containing TO are smaller than for the corresponding SPC binders themselves – dark green bars on histogram **(b)** in Figure 5. The found reduction of these values for the composites containing TO probably result from their lower workability during preparation, which affects the quality of the final monoliths [34]. In the case of the composites containing DCPD, the above values are slightly higher than for the SPCs with the DCPD modifier – brown bars on histogram **(b)** in Figure 5.

For the composites containing around 50% w/w of waste **II,** the reference sample was not available. The compressive strength for the monoliths with 2–8%sm of TO ranged from to14.8–28.4 MPa (dark green bars on histogram **(c)** in Figure 5) and increased nearly linearly with the dose of modifier. Moreover, they were approximately 20% higher when compared to the corresponding monoliths containing 25% w/w of waste **I**. In this context, a similar further increase in the compressive strength for the corresponding composites containing DCPD was found, which ranged from 14.8 to 22.8 MPa (brown bars on histogram **(c)** in Figure 5). These data for the corresponding samples containing the blended TO + DCPD sulfur modifier were irregularly scattered at an intermediate level (blue bars on histogram **(c)** in Figure 5).

The data presented above showed that despite the more difficult workability of the samples based on TO, their compressive strength was higher when compared to the respective DCPD based samples.

### 3.6 Encapsulation of galvanic wastes – immobilization of heavy metals on leaching tests

The effectiveness of encapsulation of the hazardous galvanic wastes by the SPCs was evaluated by the leaching tests' data according to TCLP and EN procedures carried out on the solidified composites. The data provided information on the immobilization of heavy metals and indicated the potential environmental impact if such materials were deposited in a landfill. The immobilization effectiveness of heavy metals from composites containing galvanic waste **I** was assessed for Cr, Cu, Zn, Ni, Pb, Cd and Sr (Table 3), while from composites containing galvanic waste **II,** it was only assessed for Cr and Zn (Table 4). The most relevant parameter determining the leachability of metals from composite matrices is their structural integrity after the encapsulation of the studied wastes (loadings around 25% w/w of waste **I** and 50% w/w of waste **II**). Other important factors are the chemical and physical characteristic of the waste, the concentration of toxic metals contained therein, the form and size of the crashed monolith grains, and the leachate equilibrium pH of the leaching test. Therefore, the effectiveness of metal immobilization in both leaching tests was expressed by a percentage of metal leachability reduction (PR) according to Eq. (2), which takes into account the dilution factor of galvanic waste by the SPC binder and its loading [35]. Calculations of PR were performed for each metal and monolith.

(2) $$PR=\left[1-(1+Additives\;Ratio)\times \frac{Concentration\;\;of\;metal\;in\;\;eluate\;from\;\;monolith\;\;composite}{Concentration\;\;of\;metal\;in\;\;eluate\;from\;\;raw\;galvanic\;waste}\right]\times 100\%$$

where $Additives\;Ratio=\frac{Mass\;of\;sulfur\;polymer\;cement\;}{Mass\;of\;dry\;galvanic\;waste\;}$

**Table 3:** Concentrations of metal ions in the TCLP and EN test leachates from the monoliths with galvanic waste **I**.

Assigned sample symbol	Concentration (mg/L)							pH	Percent Reduction (%)						
	Cr	Cu	Zn	Ni	Pb	Cd	Sr		Cr	Cu	Zn	Ni	Pb	Cd	Sr
**TCLP leachate**															
S-**I**25	24.2	47.8	44.5	2.54	0.256	0.035	0.087	3.15	-30.3	63.8	78.1	20.7	-517	73.9	75.8
ST2-**I**25	11.4	19.5	16.7	1.35	0.126	0.015	0.065	3.48	38.5	85.2	91.8	57.7	-204	88.7	81.8
ST4-**I**25	9.87	15.6	13.7	1.06	0.087	0.009	0.046	3.11	46.9	88.2	93.3	66.9	-110	93.2	87.1
ST6-**I**25	6.40	9.52	9.02	0.697	0.041	0.004	0.030	2.83	65.6	92.8	95.6	78.3	0.8	97.0	91.6
ST8-**I**25	5.20	8.34	7.96	0.579	0.022	0.003	0.024	2.82	72.0	93.7	96.1	81.9	47.1	97.7	93.2
SD2-**I**25	8.67	14.4	12.7	0.88	0.068	0.005	0.038	2.98	53.1	89.0	93.7	72.4	-64.9	96.1	89.4
SD4-**I**25	6.36	10.5	8.93	0.66	0.056	0.004	0.026	2.83	65.8	92.1	95.6	79.3	-34.8	96.8	92.8
SD6-**I**25	5.23	8.87	7.13	0.54	0.023	0.002	0.021	2.78	71.8	93.3	96.5	83.1	43.5	98.8	94.2
SD8-**I**25	4.15	6.42	5.81	0.430	0.022	0.001	0.018	2.75	77.7	95.1	97.1	86.6	47.8	99.1	95.1
**EN leachate**															
S-**I**25	0.009	0.575	10.7	0.172	nd∗	0.005	0.142	6.12	23.4	83.0	85.8	79.3	100	82.8	68.8
ST2-**I**25	0.014	0.140	2.61	0.043	nd∗	nd∗	0.040	5.81	-11.8	95.8	96.5	94.8	100	100	91.3
ST4-**I**25	0.009	0.095	2.13	0.075	nd∗	nd∗	0.054	5.92	23.8	97.2	97.2	91.0	100	100	88.1
ST6-**I**25	0.009	0.070	1.52	0.029	nd∗	nd∗	0.049	5.85	24.4	97.9	98.0	96.5	100	100	89.4
ST8-**I**25	0.005	0.039	0.353	0.002	nd∗	nd∗	0.009	5.72	56.7	98.8	99.5	99.7	100	100	98.0
SD2-**I**25	0.009	0.086	0.670	0.010	nd∗	nd∗	0.02	5.96	27.1	97.4	99.1	98.8	100	100	95.6
SD4-**I**25	0.009	0.086	0.766	0.010	nd∗	nd∗	0.019	5.95	28.4	97.5	99.0	98.8	100	100	95.7
SD6-**I**25	0.009	0.087	0.594	0.009	nd∗	nd∗	0.017	5.92	26.6	97.4	99.2	98.9	100	100	96.3
SD8-**I**25	0.009	0.083	0.605	0.009	nd∗	nd∗	0.013	5.87	26.0	97.5	99.2	98.9	100	100	97.2

∗ nd – not detected.

**Table 4:** Concentrations of metal ions in the TCLP and EN test leachates from the monoliths with galvanic waste **II**.

Assigned sample symbol	TCLP leachate					EN leachate				
	Concentration (mg/L)		pH	Percent Reduction (%)		Concentration (mg/L)		pH	Percent Reduction (%)	
	Cr	Zn		Cr	Zn	Cr	Zn		Cr	Zn
ST2-**II**50	17.9	35.2	3.10	82.7	87.8	0.060	30.2	5.08	94.2	91.0
ST4-**II**50	15.0	26.9	2.97	85.4	90.6	<0.004	11.4	5.59	99.6	96.5
ST6-**II**50	7.76	13.1	3.06	92.4	95.2	0.046	9.05	5.64	95.5	97.3
ST8-**II**50	7.29	12.2	2.95	92.7	95.5	0.012	4.39	5.83	98.8	98.7
SD2-**II**50	10.3	20.6	3.00	90.0	92.6	0.048	12.0	5.58	95.4	96.4
SD4-**II**50	7.83	16.0	2.97	92.4	94.2	<0.004	9.08	5.73	>99.6	97.2
SD6-**II**50	6.39	11.1	2.95	93.7	95.9	<0.004	7.30	5.83	>99.6	97.8
SD8-**II**50	6.72	10.9	3.03	93.3	96.0	<0.004	5.05	5.81	>99.6	98.5
S(T + D)2-**II**50	32.1	64.3	2.94	69.1	79.4	0.010	37.0	5.58	99.0	88.8
S(T + D)4-**II**50	23.3	38.9	3.26	77.5	86.9	0.063	10.1	5.31	93.9	97.0
S(T + D)6-**II**50	7.12	12.8	3.17	93.1	95.3	0.012	10.8	5.49	98.8	96.7
S(T + D)8-**II**50	8.91	14.0	2.98	91.3	94.9	0.010	7.48	5.59	99.0	97.7

**Figure 6:**
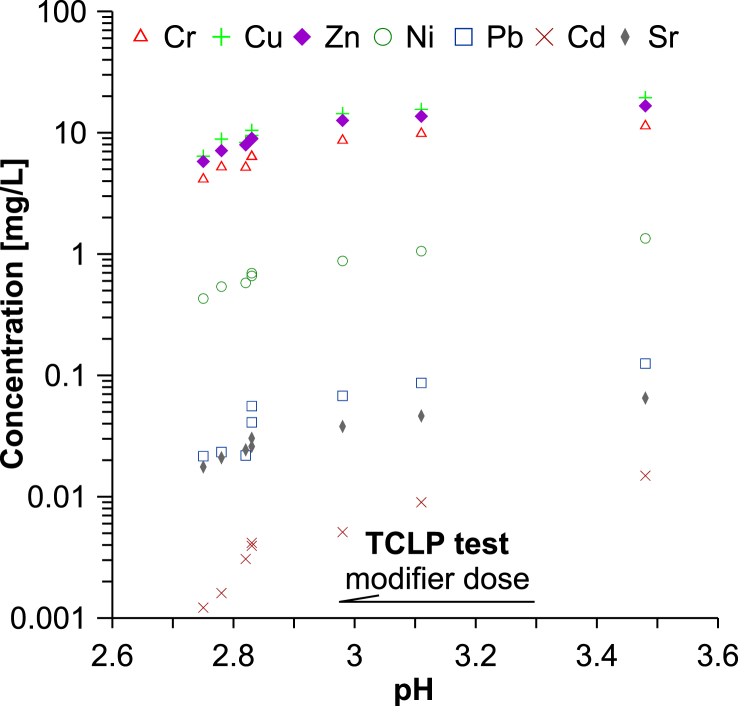
Changes of the metal ion concentrations in the TCLP test leachates as a function of pH.

The pH of the TCLP test leachates from the composites with waste **I** ranged from 2.75 to 3.48, and from the composites containing waste **II** it ranged from 2.94 to 3.26. More acidic leachate solutions promote a greater mobility of heavy metal, which is generally reflected in their higher concentrations in the TLCP test leachates. This is especially significant for Cr, for which there are compounds that are highly amphoteric (Tables 3 and 4). Generally, the higher mobilizations of Cu, Zn, Ni, Pb, Cd were assessed from the data of more acidic TCLP test leachates, than from EN test leachates, whose pH was 2–3 units higher. They were expressed by lower PR values of the metals in the TCLP test leachates when compared to the respective values in the EN test leachates. A similar PR value course was also observed for Cr in the case of composites with waste **II**, but the opposite was seen in the case of composites with waste **I.** In the case of composites with waste **I**, the TCLP test showed higher PR values than the EN test, which were of about 60% and 26%, respectively. In addition, for this metal, both leaching tests showed significantly lower PR values from the composites with waste **I** than from the composites with waste **II** (Tables 3 and 4). The reason for these inconsistencies is related to the pH of the leachates from raw galvanic wastes, and also to the high amphoteric nature of Cr compounds – their solubility decreases rapidly as the pH value rises to neutral. Despite the Cr concentration in waste **II** being more than twice as low as in waste **I**, the lower values of the leachates' pH from raw galvanic waste **II** in both leaching tests resulted in the greater leaching of this metal from waste **II** than from waste **I** (Table 1). The composites with the above wastes (25% w/w of waste **I** or 50% w/w of waste **II**) contained a comparable Cr concentration in the monoliths and also similar Cr concentration in the respective leachates from both leaching tests. In accordance to equation (2), a much higher concentration of this metal in the leachate from raw waste **II** than from raw waste **I** (Table 1) favors its higher PR value for the composite, which explains the diversity of Cr mobility. This is evidenced by the leaching tests PR values. Thus, the found effectiveness of chromium immobilization from the composites, expressed by PR values, is at level from 38 to 78% for the samples with waste **I** and from 69 to over 93% for the samples with waste **II.** These values are considered to be rather high, bearing in mind the very high concentration of this metal in the raw galvanic wastes and their high loadings into the composites (Tables 1 and 2). However, the found Cr concentration in the TCLP test leachates from the composites exceeded the regulatory limit of 5 mg/L required by the U.S. Environmental Protection Agency by a factor from 1.3 to over 6 times depending on the modifier kind used to SPC and its dose. An exception can be seen for the sample SD8-**I**25, for which the concentration of Cr in the TCLP test leachate (4.15 mg/L) did not exceed the regulatory limit. The graphical illustration of the dependence between the pH and the metal concentration in the TCLP test leachate (data in Tables 3 and 4), in relation to the increasing dose of a sulfur modifier, clearly shows its role in increasing metal immobilization. Therefore, an increased modifier dose is associated with a lowered pH and a reduction of metal concentration in the leachate. This turns out to be a common immobilization pathway for all the analyzed metals – Figure 6. The illustrated pathway is a result of increasing the crosslinking efficiency of the SPC matrix with the sulfur modifier dose. The above explanation is consistent with the observed gradual decrease in the water absorption capacity of composites with an increased dose of sulfur modifier (Figure 4).

As shown by the TCLP test, the PR values of the other most abundant metals present in the composites with waste **I** increased in the order of Ni < Cu < Zn, and were rather high in the ranges of ~50–86,6%, 63.8–95.1%, 78.1–97.1%, respectively. Their concentrations in leachate are not regulated by the federal Resource Conservation and Recovery Act (RCRA) on hazardous waste characterization. However, the slightly higher PR values were found for the composite samples containing DCPD when compared to the composite samples containing TO (Table 3). This indicates a higher waste integrity with the SPC matrix containing DCPD. The parameter discussed above also showed a continuous increase in values as the modifier dose increased. Relatively high PR values were found for Cd and Sr (>73%), and their concentrations in the TCLP test leachates were below regulatory limits (Table 3). The manifested large negative PR values for Pb, ranging from -35 to -517%, are interpreted by the amphoteric properties of its compounds. Such data result from a higher Pb concentration in the leachate of the composite samples (pH < 3.5, at which Pb mobility is much higher) than in the leachate from the raw waste sample (pH = 4.53, at which Pb mobility is significantly lower).

The leachates from the EN test had a higher pH - at the level 5.72–6.12 from the composites with waste **I,** and 5.08–5.83 from the composites with waste **II**, which significantly affected the data on the immobilization of metals from these composites. The found concentration of Cr (<0.06 mg/L) was far below the limit value of 1 mg/L that was established for the treated hazardous waste allowed for disposal at landfills assigned for depositing waste other than inert and hazardous [28]. The high PR values for Cr, which ranged from 93.9% to 99.6%, indicated its effective immobilization from the composites with galvanic wastes at a pH closer to neutral (Tables 3 and 4). The requirements of EU legislation concerning permissible limits of Zn and Ni concentration in the EN test leachates (5 mg/L and 1 mg/L, respectively) [28] were fulfilled, in case of Ni, for all the samples, while in case of Zn, the limit was only fulfilled for the one composite sample (ST8-**II**50) in which the content of TO in SPC was 8%sm. Immobilization of the Cu, Pb, Cd, and Sr expressed by the PR value were above 80%, and their concentrations in the EN test leachates from the analyzed samples (permissible limit for Cu is < 5 mg/L, for Pb is < 1 mg/L, for Cd < 0.1 mg/L, and Sr – not regulated) fulfilled the requirements of the EU legislation.

The EN test also confirmed the increased degree of metal immobilization from composites with an increased modifier dose. The influence of the kind of modifier on the effectiveness of metal immobilization is in the order: DCPD > TO > TO-DCPD.

## 4 Summary

The paper presents the evaluation of the possibility of using tung oil of biological origin as a polymerization modifier of sulfur in the preparation of SPC. Also was analyzed the use of such a cement to encapsulate of galvanic waste in order to immobilize the contained in them toxic metals: Cr, Cu, Zn, Ni, Pb, Cd and Sr. The obtained data were compared with the performance of the SPC based on the DCPD modifier and with its respective composite counterparts. The properties of SPCs modified with both sulfur modifiers and their blended mixture (TO + DCPD) in the amount of 2, 4, 6 and 8%sm each, and of monoliths with encapsulated galvanic waste with loads of around 25 or 50% w/w, were evaluated by surface morphology, water absorption by immersion, compressive strength, and in the case of composites, also by leaching tests. The evaluated data led to the following conclusions:

- FT-IR revealed the complete structural incorporation of TO triglyceride units involving double bonds in the crosslinking of the SPC matrix under conditions of its preparation,
- the monoliths' fracture surface showed a higher homogeneity for the SPC, and its composites prepared with DCPD, when compared to respective counterparts based on TO. The samples based on TO sulfur modifier contained more bubble openings with a diameter of 2 μm, and also showed lower dispersion and less effective coating of the waste grains,
- the water sorption capacity of the specimens containing TO are around twice as high as the respective specimens containing DCPD,
- a higher compressive strength was demonstrated for the SPC and composite samples prepared with TO when compared to their counterparts based on DCPD,
- the effectiveness of galvanic waste encapsulation for the immobilization of metals, assessed from both the TCLP and EN leaching tests was only slightly higher for composites prepared with the the SPC binder based on DCPD when compared to the SPC binder based on TO. The PR of the metals' leachability from the composites with encapsulated galvanic waste was very high (often far exceeded 90%), despite the high loads of wastes containing a very high concentration of toxic metals,
- an increased dose of modifier (TO or DCPD) caused a decrease in water sorption capacity, as well as an increase in the compressive strength and metal immobilization from composites with galvanic wastes.

The results presented indicate the promising practical significance of SPC based on an easily available low-cost bio-origin TO modifier that could replace currently used synthetic ones. The SPC based on TO is a competitive, high performance and environmentally friendly concurrent material when compared to other ordinary SPCs. Further extensive studies are needed to improve its preparation methodology, to determine its long term chemical resistance and mechanical performance for assessment its potential application as a construction material or binder for the disposal of hazardous wastes.

## Declarations

### Author contribution statement

Kamil Banaszkiewicz & Franciszek Czechowski: Conceived and designed the experiments; Performed the experiments; Analyzed and interpreted the data; Contributed reagents, materials, analysis tools or data; Wrote the paper.

### Funding statement

This research did not receive any specific grant from funding agencies in the public, commercial, or not-for-profit sectors.

### Competing interest statement

The authors declare no conflict of interest.

### Additional information

No additional information is available for this paper.
